# Clinical, Electrical, and Mechanical Parameters in Potassium Channel-Mediated Congenital Long QT Syndrome

**DOI:** 10.3390/jcm14082540

**Published:** 2025-04-08

**Authors:** Neringa Bileišienė, Violeta Mikštienė, Eglė Preikšaitienė, Ieva Kažukauskienė, Gabrielė Tarutytė, Diana Zakarkaitė, Rita Kramena, Germanas Marinskis, Audrius Aidietis, Jūratė Barysienė

**Affiliations:** 1 Clinic of Cardiac and Vascular Diseases, Institute of Clinical Medicine, Faculty of Medicine, Vilnius University, LT-03101 Vilnius, Lithuaniagermanas.marinskis@mf.vu.lt (G.M.);; 2 Vilnius University Hospital Santaros Klinikos, LT-08406 Vilnius, Lithuania; 3 Department of Human and Medical Genetics, Institute of Biomedical Sciences, Faculty of Medicine, Vilnius University, LT-03101 Vilnius, Lithuania; 4 Department of Pathology, Forensic Medicine and Pharmacology, Faculty of Medicine, Vilnius University, LT-03101 Vilnius, Lithuania; 5 Department of Research and Innovation, Faculty of Medicine, Vilnius University, LT-03101 Vilnius, Lithuania

**Keywords:** long QT syndrome, LQT1, LQT2, electromechanical, electrocardiogram, echocardiography

## Abstract

**Background:** Congenital long QT syndrome (LQTS) is a rare cardiac disorder caused by repolarization abnormalities in the myocardium that predisposes to ventricular arrhythmias and sudden cardiac death. Potassium channel-mediated LQT1 and LQT2 are the most common types of channelopathy. Recently, LQTS has been acknowledged as an electromechanical disease. **Methods:** A total of 87 genotyped LQT1/LQT2 patients underwent cardiac evaluation. A comparison between LQT1 and LQT2 electrical and mechanical parameters was performed. **Results:** LQT2 patients had worse electrical parameters at rest: a longer QTc interval (*p* = 0.007), a longer T_pe_ in lead V2 (*p* = 0.028) and in lead V5 (*p* < 0.001), and a higher T_pe_/QT ratio in lead V2 (*p* = 0.011) and in lead V5 (*p* = 0.005). T_pe_ and T_pe_/QT remained significantly higher in the LQT2 group after brisk standing. T_pe_ was longer in LQT2 patients compared with LQT1 patients during peak exercise (*p* = 0.007) and almost all recovery periods in lead V2 during EST. The mid-cavity myocardium mean radial contraction duration (CD) was longer in LQT2 patients (*p* = 0.02). LQT2 patients had a longer mean radial CD in mid-septal (*p* = 0.015), mid-inferior (*p* = 0.034), and mid-posterior (*p* = 0.044) segments. **Conclusions:** Potassium channel-mediated LQTS has different effects on cardiac electromechanics with a more pronounced impact on LQT2 patients. T_pe_ was more prominent in the LQT2 cohort, not only at rest and brisk standing but also during EST exercise and at recovery phases. The altered mean radial CD in the mid-cavity myocardium was also specific for LQT2 patients.

## 1. Introduction

Congenital long QT syndrome is a rare arrhythmogenic inherited cardiac disorder (ORPHA: 768). It affects voltage-gated ion channels and predisposes to polymorphic ventricular tachycardia characterized by syncope or sudden cardiac death (SCD). It is the most common and well-described cardiac channelopathy, with a prevalence of 1 in 2000 [1]. In the majority of LQTS cases, potassium ion channels are affected: the slow potassium channel *I_Ks_* (*KCNQ1* gene/LQT1) and the rapid potassium channel *I_Kr_* (*KCNH2* gene/LQT2) [2]. Electrical abnormalities reflecting pathologically dispersed prolonged myocardium repolarization in LQTS can be registered on twelve-lead ECG at rest or unmasked during provocative tests, such as brisk standing and exercise stress tests. For many years, LQTS was classically known as a ‘purely electrical’ disease. Still, evidence of LQTS-associated global and regional (subclinical) ventricular mechanical abnormalities is mounting, and the term ‘electromechanical reciprocity’ has emerged [3]. The electromechanical window of electrical–mechanical interaction in LQTS was validated for risk stratification in patients with LQTS [4,5]. Several studies of speckle tracking echocardiography (STE) explored a prolonged contraction duration (CD) and increased regional and transmural mechanical dispersion in patients with LQTS [6,7], but the data are limited.

Lots of previous studies have focused on either electrical or mechanical LQTS parameter alterations [6,7,8,9,10,11,12]. However, to our knowledge, none have provided a comprehensive evaluation of such a variety of electrical and echocardiographic parameters. Therefore, we aimed to investigate the differences in electrical and mechanical parameters between potassium channel-mediated LQTS.

## 2. Materials and Methods

### 2.1. LQTS Mutation Carriers

All patients were consulted by cardiologist in the Centre of Cardiology and Angiology of Vilnius University Hospital Santaros Klinikos between December 2022 and June 2024. Inclusion criteria were LQTS patients (≥18 years) harboring single heterozygous pathogenic or likely pathogenic *KCNQ1* or *KCNH2* gene variant. Genetic alterations were identified using next-generation sequencing in probands or Sanger sequencing in their relatives during cascade screening. Data on study population genetic variants are summarized in Table 1.

### 2.2. Clinical Evaluation

The clinical evaluation comprised detailed patient interviews, electrocardiograms, echocardiography, and laboratory test result collection. Patients were asked if they experienced severe palpitations, presyncope, syncope, or aborted cardiac arrest. Data on family history consisted of seizures in family members, syncope in 1st-degree relatives, SCD in 1st-degree relatives, or LQTS diagnosis in relatives. TSH and serum electrolytes were measured to exclude secondary QT prolongation.

### 2.3. Resting ECG

A twelve-lead ECG at a speed of 25 mm/s was recorded for each individual at rest in the supine position. Three (or less if the quality was insufficient) RR-QT couples were measured in lead II for corrected QT interval calculation. The tangent technique was used to define the end of the T-wave. Corrected QT values were calculated using the Bazett (QTcB = QT interval/(RR interval)^1/2^) formula. An electrocardiographically manifesting (penetrant) LQTS phenotype was defined as QTc value on the presenting ECG in a supine position > 460 ms [13]. We calculated the interval between the peak and the end of the T wave measured using tangent technique (T_pe_) in leads V2 and V5. We also calculated the T_pe_/QT ratio in leads V2 and V5 [9,14] as the ratio of T_pe_ to the corresponding QT interval in that lead. All these ECG parameters were measured in three consecutive beats, and a mean value was used for further analysis. Transmural heterogeneity in the LV anterior wall (ΔT_pe_), as a difference between T_pe_V5 and T_pe_V2 (T_pe_V5-T_pe_V2), was also calculated [8]. QT dispersion or inter-lead variability was calculated as a difference between the longest and shortest values of the QT interval (QT_max_-QT_min_) in each of the 12-ECG leads. QT dispersion < 50 ms was considered normal [15].

### 2.4. Brisk Standing ECG

The same parameters as resting supine twelve-lead ECGs were measured and calculated on twelve-lead ECGs after standing briskly. A brisk standing ECG was made from resting supine, moving quickly to the orthostatic position [16]. A swift of 20–30 beats per minute (bpm) increase in a person’s sinus rate was expected to result in changes in the QTc interval [17]. Paradoxical QTc prolongation > 30 ms was considered abnormal [17,18].

### 2.5. Exercise Stress Test

All patients underwent a standardized diagnostic exercise stress test (EST) using a cycling ergometer. Twelve-lead ECG was registered at rest (seating position) during each phase of the exercise (a serial rise in 50 W every 3 min) and at 1 min intervals during the 7 min of the recovery phase. The peak of exercise ECG was considered as a twelve-lead ECG written at submaximal heart rate. In each ECG, QTcB, T_pe_, and T_pe_/QT ratios were calculated as described in Section 2.3.

### 2.6. Echocardiographical Data

All individuals underwent conventional 2-dimensional (2-D) echocardiography to assess left ventricular (LV) function and exclude structural heart disease. Images at high frame rates (>60 frames per second) were used for LV strain analysis with EchoPAC (GE Healthcare). LV global longitudinal strain was analyzed in 3 apical LV-focused views (apical long-axis view, 4-chamber view, and 2-chamber view) using the two-dimensional strain speckle-tracking application in a standard 18-segment model [19]. The time from ECG R wave peak to maximum myocardial shortening was defined as contraction duration (CD). Longitudinal and radial CDs of 18 LV segments were measured from apical and parasternal short-axis views, respectively. The longitudinal and radial strain was subdivided into basal, mid, and apical planes (6 segments in each plane). The standard deviation of the 18 longitudinally and radially measured CDs was calculated as parameters of longitudinal and mechanical dispersion.

Electromechanical window (EMW) was calculated as the time difference between the interval from QRS onset to aortic valve closure (midline) in the continuous-wave Doppler images in the apical long-axis view and QT interval for the same beat from the ECG curve [4].

Apical 4- and 2-chamber view images of the LA using conventional 2-D echocardiography at high frame rates (>60 fps) were utilized for LA reservoir strain (LARS) analysis. The left atrial volume index (LAVI) was calculated from LA volume (biplane) and body surface area ratio. Isovolumic relaxation time (IVRT) was obtained from pulsed Doppler images showing both aortic outflow and mitral inflow. As parameters of diastolic dysfunction, IVRT values < 60 or >90, LAVI > 34 cm/m^2^, or LARS < 18 were considered abnormal. Patients older than >60 years or those who were diagnosed with arterial hypertension were excluded from the evaluation of diastolic function.

### 2.7. Statistical Analysis

Statistical analysis was performed using R software (version 4.2.2, R Foundation for Statistical Computing, Vienna, Austria). Continuous variables were tested for normal distribution using the Shapiro–Wilk test. Normally distributed continuous variables were expressed as the mean ± standard deviation. Other continuous variables were expressed as the median and IQR, categorical data as counts and percentages.

Continuous variables were compared using Student’s independent t-test when normally distributed. The Wilcoxon rank sum was used for the comparison of not normally distributed data. Categorical values were compared using the chi-square test or Fisher’s exact test if expected values were <5. Two-tailed *p* values ≤ 0.05 were considered significant.

Receiver operating characteristic (ROC) was performed, and the area under the ROC curve (AUC) was calculated for the parameter to estimate how well they can differentiate between LQT1 and LQT2 groups. Sensitivity and specificity for the optimal cut-off value were reported to distinguish the parameters between LQT1 and LQT2 groups.

### 2.8. Ethical Approval and Informed Consent

This study was approved by The Vilnius Regional Biomedical Research Ethics Committee (approval code: 2020/1-1182-669). All participants gave their written informed consent for inclusion. The investigation was performed by following the Helsinki Declaration.

## 3. Results

### 3.1. Clinical Evaluation

A total of 87 genotyped LQTS patients were included in the study. LQT1 and LQT2 patients did not differ by age, gender, heart rate, and blood pressure measurements. Variant penetration in ECG was also similar between groups (20.0% in LQT2 vs. 14.5% in LQT1, *p* = 0.5). LQT2 patients experienced symptoms such as premature ventricular contractions (44.0% vs. 19.4%, *p* = 0.018), presyncope (48.0% vs. 17.7%, *p* = 0.004), or syncope (32.3% vs. 68.0%, *p* = 0.002) more frequently compared with LQT1 patients. Syncope in the 1st-degree family members was also more frequent in the LQT2 group (52.0% vs. 19.4%, *p* = 0.09). Patients` clinical data are presented in Table 2.

### 3.2. Resting ECG

All patients were in sinus rhythm. Twelve patients were treated with an implantable cardioverter defibrillator, but none had ventricular pacing during their clinical evaluation. Patients on beta-blockers during clinical examination were excluded from all ECG (including brisk standing and EST) data analysis.

**Figure 1:**
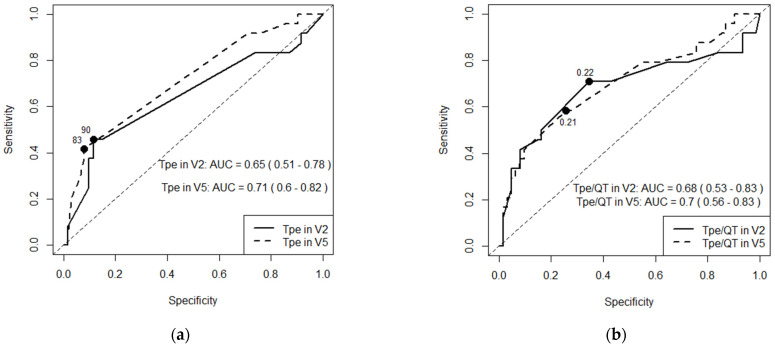
ROC curves for (**a**) T_pe_ in ms and (**b**) T_pe_/QT values in precordial V2 and V5 leads.

LQT2 patients had longer QTcB interval (440 ± 29 ms vs. 419 ± 37 ms, *p* = 0.007). Wilcoxon rank sum test showed higher transmural heterogeneity in LQT2 patients: this group had longer T_pe_ (80 (80, 118) ms vs. 80 (78, 80) ms, *p* = 0.028) in leads V2 and V5 (80 (80, 107) ms vs. 80 (70, 80) ms, *p* < 0.001) and higher T_pe_/QT ratio (0.2 (0.2, 0.3) vs. 0.2 (0.2, 0.2), *p* = 0.011) in leads V2 and V5 (0.2 (0.2, 0.3) vs. 0.2 (0.2, 0.2), *p* = 0.005). In ROC curve analysis of T_pe_ and T_pe_/QT ratio, T_pe_ performed best in lead V5 with an AUC of 0.71 (95% CI 0.60 to 0.82) at a value of 90 ms as a cut-off between LQT2 and LQT1 groups (Figure 1).

We did not find a significant difference in calculating transmural heterogeneity in the LV anterior wall (ΔTpe) between these two groups. QTc dispersion (inter-lead variability) >80 ms was only observed in 3 (12.0%) patients in the LQT2 group; the rest of the study population had a normal inter-lead repolarization dispersion.

### 3.3. Brisk Standing Test

After brisk standing, the QTcB interval did not significantly differ between groups: in LQT2, the median QTcB was 458 (436.4, 464.8) ms, and in LQT1, it was 433 (415.5, 467.2) ms, *p* = 0.132. Other parameters that differed between groups in resting ECG unchanged after brisk standing: we still observed higher transmural heterogeneity in the LQT2 group in lead V2 (80 (77, 120) ms vs. 80 (67, 80) ms, *p* = 0.033) and in lead V5 (80 (80, 118) ms vs. 80 (69, 80) ms, *p* = 0.015), as well as corrected Tpe/QT in lead V2 (0.2 (0.2, 0.3) vs. 0.2 (0.2, 0.2), *p* = 0.026) and in lead V5 (0.2 (0.2, 0.3) vs. 0.2 (0.2, 0.2), *p* = 0.013). Paradoxical QTc prolongation was not associated with the LQTS genotype in our study population.

### 3.4. Exercise Stress Test

**Figure 2:**
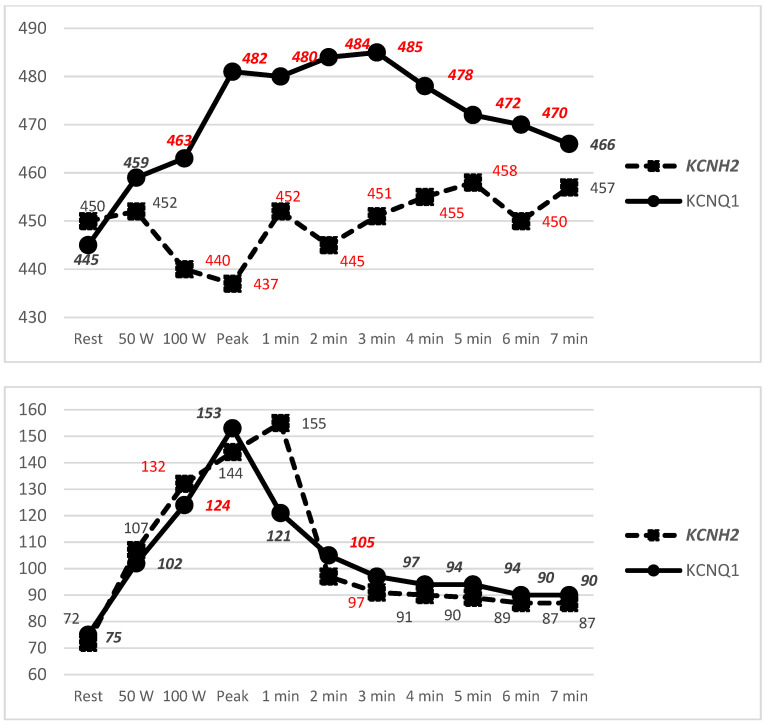
Mean QT intervals using the Bazett formula (ms) [**top**] and mean heart rate (bpm) [**under**] plotted against time during EST. Values marked in red differed significantly (*p* ≤ 0.05).

At rest in the supine position, the twelve-lead ECG heart rate between the groups was similar, and QTcB was similar between LQT1 and LQT2 patients. The difference in HR and QTcB values became evident at the 100 W stage, where LQT2 patients developed a higher HR (*p* = 0.01) but had a shorter QTcB interval (*p* = 0.024) compared with LQT1 patients. In LQT2, the mean HR was 132 ± 21 bpm, QTcB 440 ± 32 ms, and in the LQT1 group, the mean HR was 124 ± 20 bpm, QTcB 463 ± 43 ms. LQT1 patients had longer QTcB intervals at the EST peak exercise stage: LQT1 patients’ QTcB was 482 ± 56 ms, and that of LQT2 patients was 437 ± 34 ms, *p* = 0.016. The difference in the QTcB duration between the LQT1 and LQT2 groups was observed up to the 6th minute of the recovery stage (Figure 2).

**Figure 3:**
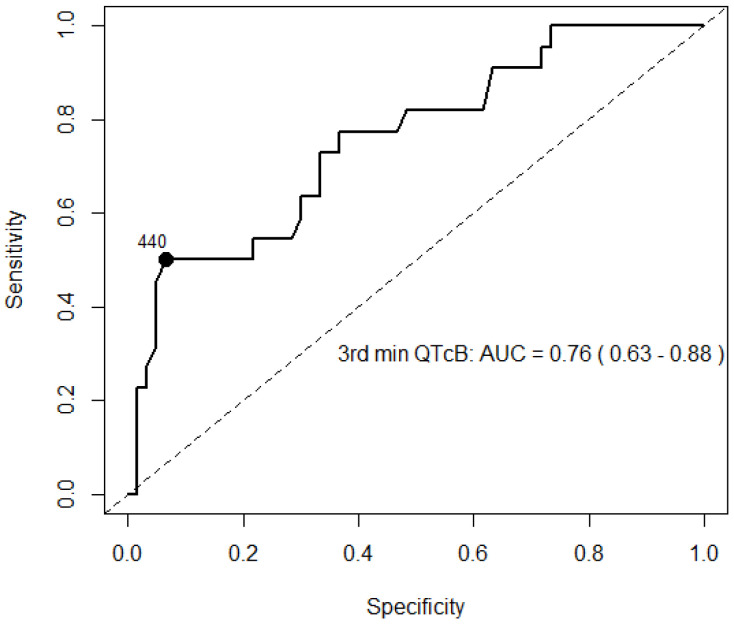
ROC curve for QTcB (ms) during 3rd minute of recovery during EST.

ROC analysis revealed the ability to differentiate LQT1 and LQT2 types, with the highest specificity and sensitivity at the 3rd minute of recovery (Figure 3).

The Wilcoxon rank sum test showed that higher LQT2 patients lead V2 T_pe_ values in comparison to LQT1 patients (67 (57–80) ms vs. 47 (40–60) ms; *p* = 0.007) in terms of the peak exercise stage. We did not observe this in lead V5, as it was on resting ECG or ECG after brisk standing.

**Figure 4:**
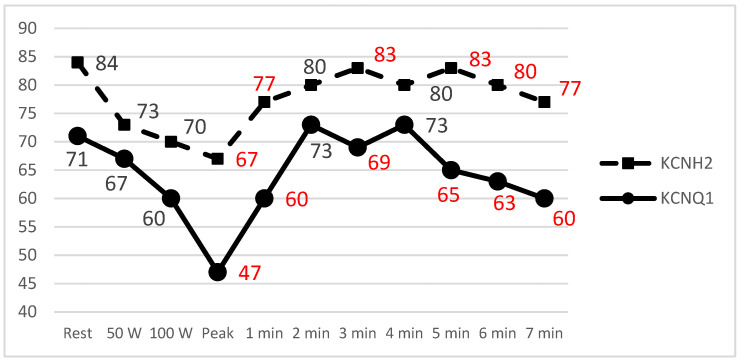
Mean T_pe_ plotted against time during EST. Values marked in red differed significantly (*p* ≤ 0.05).

We observed longer T_pe_ values for LQT2 patients in precordial lead V2 for almost the entire recovery period (except for the 2nd and 4th minute). The longest T_pe_ values were observed in LQT2 patients during the 3rd and 5th minutes of the recovery phase; they were 83 ± 29 ms and 83 ± 32 ms, accordingly (Figure 4).

### 3.5. Echocardiographical Findings

**Figure 5:**
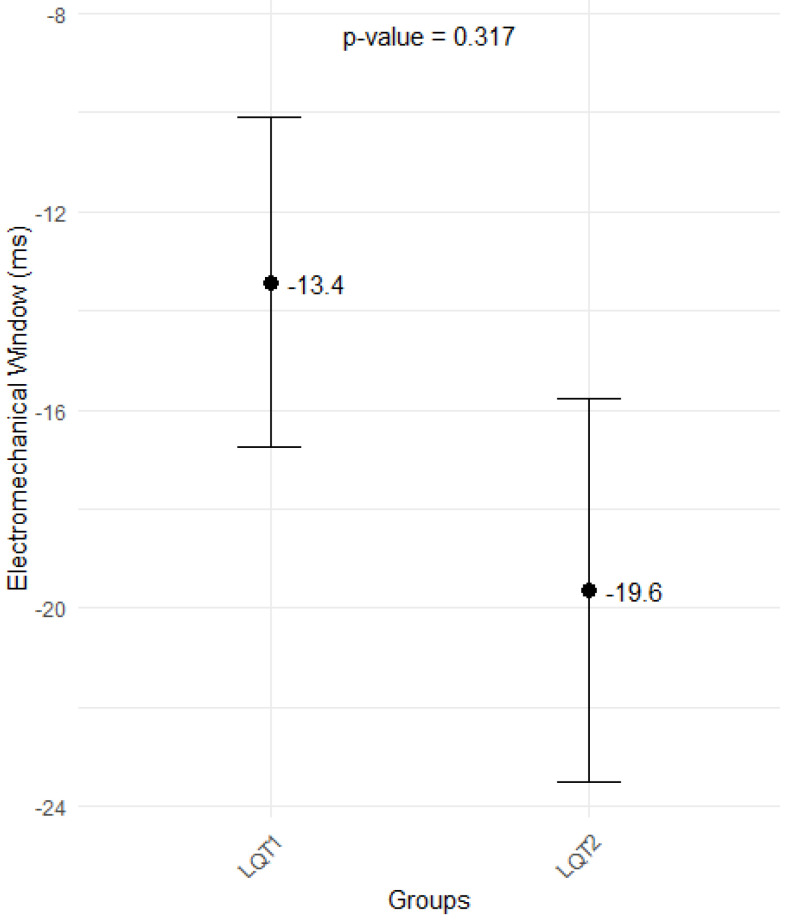
Box plots showing difference in electromechanical window between patients in LQT1 and LQT2.

The parameter of LV systolic performance—global longitudinal strain (GLS)—was within normal limits and did not differ between LQT2 and LQT1groups (−21.0 ms ± 1.7 vs. 20.8 ± 1.9, *p* = 0.766), with EMW also in absolute values (Figure 5). In both groups, most of the patients had negative EMW: 18 (81.8%) LQT2 patients and 39 (69.6%) LQT1 patients, *p* = 0.28.

In the study population, we observed sporadic abnormal LV diastolic function parameters, but no differences were found between the groups of LQT2 and LQT1: their IVRT was 76 vs. 73 ms, *p* = 0.7, their LARS was 38 vs. 37%, *p* = 0.606, and their LAVI was 24 vs. 24 cm/m^2^, *p* = 0.94.

We did not observe a difference between the two groups in terms of the mean longitudinal or radial contraction durations and longitudinal or radial mechanical dispersions. When the LV myocardium was divided into three planes (basal, mid-cavity, and apical), the median mid-cavity myocardium CD was observed to be longer in LQT2 patients (376 (362, 430) ms vs. 362 (342, 382) ms; *p* = 0.02), with a tendency to median mid-cavity myocardium mechanical dispersion (10 (6, 34) ms vs. 7 (0, 13) ms *p* = 0.06). Subgroup LV segmental analysis showed more extended radial CD in LQT2 patients in the mid-cavity (papillary muscle level) septal (377 (357, 431) ms vs. 359 (335, 375) ms, *p* = 0.015), inferior (378 (358, 429) ms vs. 363 (338, 380) ms, *p* = 0.034), and posterior (380 (360, 448) ms vs. 364 (343, 392) ms, *p* = 0.044) LV segments in comparison with LQT1 patients.

## 4. Discussion

In this cross-sectional study, we aimed to investigate the differences in electrical and mechanical parameters between our population of LQT1 and LQT2 patients during a comprehensive evaluation of their clinical, electrical, and mechanical variables. To our knowledge, this is the first attempt to assess established genotype-specific parameters and search for new genotype-specific parameters in LQTS patients when analyzing their resting ECG, provocative test (brisk standing and exercise test with long recovery period) results, and echocardiographic data in the same patient cohort. Previous studies focused on either ECG, provocative tests, or echocardiographic data with a few parameters from a different diagnostic tool.

It is well established that congenital LQTS most manifests commonly as a syncope [20,21], but the rate varies in studies from 26% to 50%. Moening et al. [22] reported syncope in 58% of LQT2 patients vs. 41% in LQT1 patients, and Kutyifa et al. reported [23] 37 vs. 36%, respectively. In our study population, 68% of LQT2 patients had experienced syncope (assumed as arrhythmic), while in the LQT1 cohort, 32% had such an episode.

Prolongation of the QTc (QT) interval is a hallmark of the disease due to the imbalance of delicate ion currents in the repolarization phase in the ventricular action potential. In our cohort, the electrocardiographically manifest (penetrant) LQTS phenotype did not differ between the groups and was low; overall, LQT2 patients had longer QTc values in terms of the resting ECG. However, there is inconsistency in data on the QTc length in different LQTS types between studies [2,8,24], and the length of the QTc has never been a discriminator between LQTS types.

Another altered (proarrhytmogenic) ECG parameter observed in LQTS patients, known as a marker of global spatial dispersion of repolarization, is T_pe_ [3]. It reflects the dispersion of repolarization in ventricular layers and does it more accurately than the QT interval [25]. The T_pe_ interval can be used as a diagnostic criterion in differentiation between LQT2 and LQT1 patients [26,27]. Rieder et al. found significantly prolonged T_pe_ in lead V2 but not in lead V5 in patients with LQT2 [8]. Meanwhile, in our larger than previously investigated LQT2 cohort, we found T_pe_ prolonged in both precordial leads (V2 and V5). Moreover, ROC curve analysis showed that T_pe_ in V5 performed better than in V2. Rieder et al. investigated the ΔT_pe_ parameter, calculated as a difference between T_pe_ V5 and T_pe_ V2, and found it more negative in LQT2 than in LQT1 [8]. In our study, LQT2 patients had more prominent T_pe_ and a higher T_pe_/QT ratio, but we did not observe a difference in ΔT_pe_ among the LQT1 and LQT2 groups.

QT dispersion (inter-lead variability) in a standard twelve-lead ECG is considered an indirect measure of spatial heterogeneity of repolarization in the myocardium and reflects inhomogeneity of ventricular action potentials. QT dispersion values of 65 ms or greater carry a high risk of ventricular arrhythmias and are a risk marker for SCD [28]. QT dispersion was found to increase in patients with LQTS, especially in those with an arrhythmic phenotype [8]. In our study, only several LQT2 patients had significant QT dispersion of 80 ms, and all of them had an evident arrhythmic phenotype.

Maladaptive repolarization manifesting during autonomic nervous system changes is suggested to be a provoked feature that differentiates healthy individuals from LQTS patients. Unfortunately, HR changes during provocative tests are not always reached. In our cohort, LQTS patients also failed to reach the expected HR change, but the LQT2 group had higher HR compared to the LQT1 group. This might partly relate to the fact that patients with LQTS, particularly LQT1, have demonstrated a primary sinoatrial node phenotype of chronotropic insufficiency in previous studies [29]. Aziz et al. showed that postural changes in the QT interval were not helpful in discriminating between LQT1 and LQT2 genotypes, as a blunted heart rate acceleration was observed in LQTS compared with the control group [24]. We also did not observe a difference in the postural change to the QTc interval between groups. We only observed that the QTc interval became similar between the groups during the brisk standing test, probably because of underlying altered cellular mechanisms in LQT1 patients. Longer T_pe_ and a higher T_pe_/QT ratio remained in LQT2 patients after brisk standing. Predominant prolongation of the M-cell action potential —leading to increased transmural dispersion of repolarization (TDR) and early after depolarization activity—is a well-recognized response to heart rate acceleration in models of LQT2 [18].

The exercise stress test in LQTS allows for an extended assessment of QT and T-wave changes during both HR acceleration and deceleration phases [30]. Changes in the QTc interval during exercise and recovery are helpful in distinguishing between LQTS genotypes. It is postulated that differences in QTc interval duration during peak exercise or at early (1 min) to mid-recovery phase (3–4 min) could help differentiate LQT1 and LQT2 patients [17,30,31]. Our study data also showed the ability to differentiate LQT1 and LQT2 types at peak exercise and the early recovery phase (at the 3rd minute) of EST. This explains that predominant cellular repolarization currents become of great importance at critical heart rates: the *KCNQ1* gene encodes the *I_Ks_* channel, and the absence of functional *I_Ks_* results in paradoxical QTc prolongation at fast heart rates (during peak exercise and the early recovery phase). Meanwhile, impairment of the *KCNH2* gene encoded delayed rectifying current *I_Kr_* channel (LQT2 patients) becomes more evident at intermediate heart rates [11]. Consequently, during peak exercise and recovery phases, when the heart rate remains relatively fast, LQT2 patients have normal QTc adaptation and minimal QTc prolongation [31]. LQT1 patients are known to have a sharp increase in the QT interval during exercise, followed by a gradual normalizing of the QT interval during the recovery phase. Meanwhile, LQT2 patients exhibit a typical repolarization pattern during exercise, but the QTc interval lengthens during the recovery period [17]. Hekkala et al. reported that during the EST, in LQT1 patients, the T_pe_ interval did not shorten during effort [32]. Meanwhile, LQT2 patients have a longer T_pe_ interval than other subtypes at a low HR and are known to show proper shortening of QT and T_pe_ intervals during effort. In our cohort, LQT2 patients had prolonged T_pe_ intervals from the peak exercise phase almost permanently to the 7th minute post-exercise. The shortest T_pe_ values in LQT2 patients were observed at the peak of exercise but remained significantly different between the groups. Experimental data might explain this finding. It is known that beta-adrenergic stimulation with isoproterenol transiently prolongs action potential durations in M cells, possibly because of a more rapid increase of inward *I_Na-Ca_* current than of outward *I_Ks_* current and LQT2 patients who have already altered M cell action potential due to the delayed rectifying current *I_Kr_* channel dysfunctional exhibiting more evident TDR [27,33]. To our knowledge, we described a gene-specific comparison of a 7 min long recovery period of this recognized T_pe_ parameter for the first time. Aziz et al. investigated a nine-minute recovery period in a population less than 21 years old, but they concentrated on QT and QTc interval changes [24].

Echocardiography is mostly used to exclude structural heart disease when LQTS diagnosis is based on phenotype features without pathogenic variants in LQTS-related genes. Sporadic but consistent evidence that electric alterations in LQTS patients can have mechanical consequences has been accumulating in the past 20 years [6]. Now, there are convincing data that mechanical consequences of *I_Ks_* and *I_Kr_* ion channel dysfunctions, named electromechanical reciprocity, exist [3]. Heterogeneity of repolarization may contribute to or reflect abnormal myocardial mechanics due to a heterogeneous myocardial contraction duration [34]. Echocardiographic strain imaging demonstrated this phenomenon [6,7]. More prominent mechanical alterations in subjects with *I_Kr_* channel dysfunction (genotype-specific) are described [2]. We observed the difference in the radial mean contraction duration and the tendency for mechanical dispersion in the mid-cavity myocardium (papillary muscle level) in the LQT2 patients’ group. Subsegmental analysis of mid-cavity LV myocardium showed a difference in the mean radial CD in septal, inferior, and posterior LV segments. Radial strain analysis in the LQTS cohort was performed by Borowiec et al. [6]. Still, they did not provide data on LQT1 and LQT2 patients’ comparison and mid-cavity myocardium radial strain performance. Their principal findings were that myocardial CD is prolonged for both radial and longitudinal directions in LQTS patients, and they found an association between apical radial deformation and subjects at higher risk of arrhythmic events. Haugaa et al. did not report significant differences between LQT1 and LQT2 patients in terms of the mean longitudinal CD or mechanical dispersion [7], which aligns with our study findings. In segmental LV strain analysis between LQT1 and LQT2, Haugaa et al. revealed prominent transmural mechanical dispersion in LQT2 patients in the posterior septal, anterior septal, and anterior segments. Transmural mechanical dispersion was not present in asymptomatic LQT2 mutation carriers [7]. We could not perform transmural dispersion analysis as we did not perform circumferential strain analysis. Still, we found more prolonged radial contraction durations, which reflect longer systolic myocardium thickening [35], in LQT2 patients compared with LQT1 patients in LV mid-septal, mid-inferior, and mid-posterior LV segments. Interestingly, regional differences in the repolarization time are seen in these regions of the human heart [26]. The interventricular septum is known to have M cells in the deep subendocardium, which strongly influence APD prolongation in neighboring endocardial cells [7]. The investigators hypothesized that this finding might indicate genotype-specific differences in electric dispersion and are in accordance with the higher arrhythmic risk in patients with LQT2 and JLNS. Contraction abnormalities in LQTS patients may lead to subclinical impairment of myocardial function. It was observed that patients with LQTS have subtle systolic dysfunction, which is objectivized by measuring GLS [2,7]. We report the same finding. Despite a normal LV size and ejection fraction, the GLS, which reflects subendocardial function, is reduced in LQTS patients. This might be related to the Purkinje fiber conduction system embedded in the subendocardial myocardium layer. Purkinje fiber repolarization abnormalities play a significant role in LQTS arrhythmogenesis [36]. Diastolic dysfunction was also shown to be found in LQTS patients [2], but in our cohort, we observed only sporadic abnormal diastolic dysfunction parameters (IVRT, LAVI, and LARS) in the LQT1 or LQT2 patient cohorts. To our knowledge, LARS has never been evaluated as a diastolic dysfunction parameter in LQTS patients, but it did not differ between groups.

Another validated electromechanical parameter in patients with LQTS is EMW [4], caused by disproportionately prolonged repolarization, which outlasts mechanical systole. In our LQTS cohort, no differences were observed between the two LQTS types in accordance with Sugrue et al.’s study [4]. Still, in both groups, for the majority, it was negative.

## 5. Limitations

The findings from our study are limited due to the small number of participants, as this was a single-center study in Lithuania. However, phenotype assessment was conducted by the same LQTS specialist. This minimized the heterogeneity typically introduced by clinical evaluations carried out by multiple specialists, even among the experts in the field. As it was a cross-sectional study, we could not report the impact of electromechanical parameters on the later course of the disease. Also, we hope for larger prospective studies to be conducted to uncover the effect of beta-blockers on the changes in QT intervals, Tpe, and diastology.

## 6. Conclusions

Potassium channel-mediated LQTS have different consequences for cardiac electromechanics. Transmurally dispersed repolarization is more evident in LQT2 patients during all phases of EST. An altered mean radial contraction duration in mid-cavity myocardium is also type-specific, particularly in the septal segment, for patients with LQT2.

## Figures and Tables

**Table 1:** Summary of genetic variants in the study population.

Gene	Mutation Type	Nucleotide Change	No. of Participants
*KCNQ1* (NM_000218.3)	Missense	c.701A>C	1
	Missense	c.1831G>A	1
	Missense	c.568C>T	1
	Missense	c.1111G>C	9
	Splicing	c.477+1G>A	34
	Deletion	c.1265del	8
	Missense	c.502G>A	1
	Missense	c.674C>T	2
	Deletion	c.790delA	3
	Nonsense	c.513C>A	2
*KCNH2* (NM_000238.4)	Missense	c.243G>C	2
	Deletion	c.321_322del	2
	Missense	c.526C>T	2
	Deletion	c.681delC	2
	Missense	c.859G>T	1
	Missense	c.1141G>T	1
	Missense	c.1600C>T	6
	Missense	c.1750G>A	2
	Missense	c.1832A>T	1
	Missense	c.1849T>G	1
	Missense	c.1891T>C	2
	Missense	c.2453C>T	2
	Deletion	c.3103_3152+6del	1

**Table 2:** Clinical characteristics of LQTS mutation carriers.

	LQT1 (n = 62)	LQT2 (n = 25)	*p* Value
Age, y	37.0 (23.0, 44.8)	40.0 (28.0, 46.0)	0.6
Female, n (%)	36 (58.1%)	17 (68.0%)	0.4
Probands, n (%)	21 (33.9%)	11 (44.0%)	0.4
HR, bpm	72 (64, 78)	73 (65, 83)	0.8
Hypertension, n (%)	10 (16.1%)	5 (20.0%)	0.8
Systolic BP, mmHg	130 ± 15	127 ± 12	0.3
Diastolic BP, mmHg	83 ± 11	82 ± 8	0.6
Premature ventricular contractions, n (%)	12 (19.4%)	11 (44.0%)	0.02
Palpitations, n (%)	13 (21.0%)	9 (36.0%)	0.1
Penetration ECG, n (%)	9 (14.5%)	5 (20.0%)	0.5
Syncope, n (%)	20 (32.3%)	17 (68.0%)	0.002
Presyncope, n (%)	11 (17.7%)	12 (48.0%)	0.004
Aborted cardiac arrest, n (%)	1 (1.6%)	2 (8.0%)	0.2
Ventricular tachycardia, n (%)	3 (12.0%)	1 (1.6%)	0.07
SCD in 1st-degree relatives, n (%)	17 (27.4%)	8 (32.0%)	0.7
Syncope in 1st-degree relatives, n (%)	12 (19.4%)	13 (52.0%)	0.01
Seizures in family members, n (%)	3 (4.8%)	3 (12.0%)	0.3
LQTS in relatives, n (%)	40 (64.5%)	14 (56.0%)	0.5
BNP, ng/L	12.5 (0.0, 19.2)	14.0 (0.0, 23.0)	0.2
Potassium, mmol/L	4.5 (4.3, 4.7)	4.6 (4.3, 4.8)	0.5
Without beta-blocker during clinical evaluation, n (%)	55 (89%)	25 (100%)	0.2

BNP—brain natriuretic peptide, BP—blood pressure, ECG—electrocardiogram, HR—heart rate, LQTS—long QT syndrome, SCD—sudden cardiac death.

## Data Availability

The data presented in this study are available upon request from the corresponding author, N.B.
